# Clinical significance of pre-surgical serum lipid levels in patients with glioblastoma

**DOI:** 10.18632/oncotarget.20730

**Published:** 2017-09-08

**Authors:** Ruofei Liang, Junhong Li, Mao Li, Yuan Yang, Xiang Wang, Qing Mao, Yanhui Liu

**Affiliations:** ^1^ Department of Neurosurgery, West China Hospital, Sichuan University, Chengdu, China

**Keywords:** pre-surgical, lipid levels, glioblastoma

## Abstract

Accumulating evidence demonstrates that pre-surgical serum lipid levels are linked to the clinical outcome of different types of human malignant tumors, but few studies have explored the prognostic value of these easily accessible parameters in glioblastoma. The aim of the current study was to validate the association between pre-surgical serum lipid levels and the clinical outcome of patients with glioblastoma. The pre-surgical serum lipid levels (triglycerides, total cholesterol, high-density lipoprotein [HDL] cholesterol, and low-density lipoprotein [LDL] cholesterol) of 125 patients with glioblastoma, who underwent surgery between January 2015 and May 2016, were retrospectively evaluated. The correlation between pre-surgical serum lipid levels and overall survival (OS) was examined using the Kaplan-Meier method and Cox proportional hazards regression model. Univariate analysis showed that lipids associated with OS were total cholesterol, HDL cholesterol, and LDL cholesterol levels. Results of multivariate analysis identified LDL cholesterol level as an independent prognostic factor for OS in patients with glioblastoma (hazard ratio: 0.412; 95% confidence interval: 0.211-0.801; P = 0.009). Total cholesterol and HDL cholesterol levels were predictive factors only in univariate analysis, but not in multivariate analysis. The current study demonstrated that pre-surgical serum LDL cholesterol level is an independent prognostic factor for clinical outcomes of patients with glioblastoma. Pre-surgical serum LDL cholesterol level might provide valuable prognostic information for patients with glioblastoma that could be applied in clinical practice.

## INTRODUCTION

Glioblastoma, the most aggressive glioma type, accounts for approximately 70% of all malignant gliomas [1]. However, the prognosis is poor even if patients receive comprehensive treatment (tumor resection, radiotherapy, and chemotherapy). The median overall survival (OS) has increased only by 3.3 months (from 11.3 months to 14.6 months) in patients diagnosed with glioblastoma [2].

Currently, many novel peripheral blood biomarkers have been identified to predict prognosis in patients with glioblastoma, and have gained popularity because of their accessibility. For instance, increased plasma insulin like growth factor binding protein 2 (IGFBP-2) levels after post-surgical adjuvant therapy (chemotherapy plus radiotherapy) in elderly patients have been linked to worse clinical outcomes [3]. Additionally, Li et al. found that a low expression level of pre-surgical serum microRNA-137 was strongly associated with poor OS in patients with glioblastoma [4]. However, the clinical utility of these biomarkers is limited because of the high detection costs and specialized diagnostic techniques required. Thus, reliable and easily detectable biomarkers in peripheral blood that correlate strongly with prognosis are urgently needed.

Growing evidence suggests that lipid metabolism is strongly associated with cancer prognosis. Many studies have reported that pre-surgical serum total cholesterol level correlate with disease prognosis in malignancies such as clear cell renal cell carcinoma, non-small-cell lung cancer, and esophageal squamous cell carcinoma [5–7]. Recently, one study reported that pre-surgical serum triglycerides and high-density lipoprotein (HDL) cholesterol levels may be independent prognostic factors in patients with breast cancer [8]. In addition, another study showed an association between high low-density lipoprotein (LDL) cholesterol level and poor disease-free survival in patients with breast cancer [9]. To our knowledge, the prognostic value of lipid levels has never been investigated in patients with glioblastoma. Therefore, the aim of the current study was to examine the relationship between pre-surgical serum lipid levels and survival of patients with glioblastoma.

## RESULTS

### Patients characteristics

In total, 125 patients with glioblastoma were included in the analysis (Table 1). There were 81 men and 44 women, and the median age was 57 years (range: 17–81). A total of 101 patients were aged <65 years and 24 patients were aged ≥65 years. At the last follow-up, 27 patients were still alive and 98 were dead. Using the X-tile software, cut-off values of 3.91 mmol/L, 1.32 mmol/L, and 1.84 mmol/L were selected for total cholesterol, HDL cholesterol, and LDL cholesterol levels, respectively. As the X-tile software was unable to select a significant cut-off value for triglycerides, the median triglycerides value (1.24 mmol/L) was used.

**Table 1:** Baseline patient characteristics

Variables	N	%
Age (years)		
≥65	24	19.2
<65	101	80.8
sex		
Male	81	64.8
Female	44	35.2
Tumor size (cm)		
≥3	116	92.8
<3	9	7.2
Tumor location		
Frontal lobe	49	39.2
Temporal lobe	50	40
Midline structure	33	26.4
Involvement of mutilobar	38	30.4
Other locations	47	37.6
Extent of resection		
Gross total	80	64.0
Subtotal	45	36.0
Adjuvant radio/chemotherapy		
Yes	59	47.2
No	66	52.8

### Correlation of pre-surgical serum lipid levels with OS

**Figure 1:**
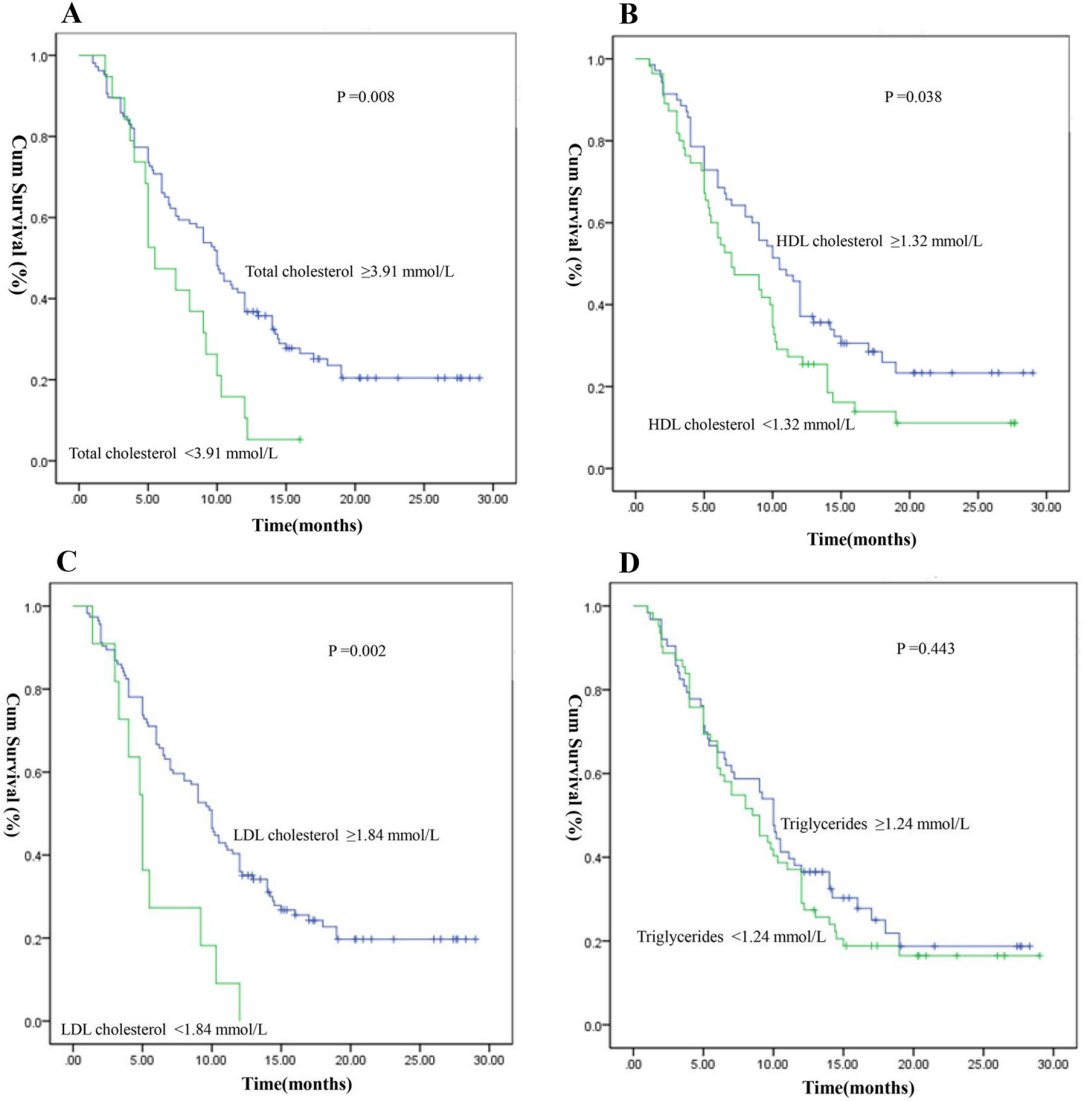
Kaplan-Meier survival curves of serum lipid levels for overall survival (OS) (**A**) OS stratified by their pre-surgical total cholesterol; (**B**) OS stratified by their pre-surgical HDL cholesterol; (**C**) OS stratified by their pre-surgical LDL cholesterol; (**D**) OS stratified by their pre-surgical triglycerides. (with log-rank test).

Kaplan–Meier survival curves for the serum lipid levels of OS according to the optimal cut-off value are shown in Figure 1. Patients with low total cholesterol level showed worse OS than those with high cholesterol level (total cholesterol level: <3.91 mmol/L vs. ≥3.91 mmol/L; median OS: 5.50 vs. 10.0 months, P =0.008) (Figure 1A). Patients with HDL cholesterol level <1.32 mmol/L had a median OS of 7.0 months, whereas patients with HDL cholesterol level ≥1.32 mmol/L presented with a median OS of 10.5 months (P =0.038) (Figure 1B). Patients with low LDL cholesterol level showed remarkably worse OS than those with high LDL cholesterol level (LDL cholesterol level: <1.84 mmol/L vs. ≥1.84 mmol/L, median OS: 5.0 vs. 10.0 months, P =0.002) (Figure 1C). Triglycerides level (<1.24 mmol/L vs. ≥1.24 mmol/L) was not significantly predictive of OS (median value: 8.5 vs. 10.0 months, P =0.443) (Figure 1D).

Univariate analysis showed that the lipid parameters associated with OS were total cholesterol, HDL cholesterol, and LDL cholesterol levels. Total cholesterol level >3.91 mmol/L, HDL cholesterol level >1.32 mmol/L, and LDL cholesterol level >1.84 mmol/L were associated with favorable prognosis. However, triglycerides level had no correlation with OS in patients with glioblastoma.

Results of multivariate analysis identified LDL cholesterol level as an independent prognostic factor for OS (hazard ratio: 0.412; 95% confidence interval: 0.211-0.801; P =0.009). The total cholesterol and HDL cholesterol levels were predictive only in univariate analysis, but not in multivariate analysis (Table 2).

**Table 2:** Univariate and Multivariate Cox proportional analysis regarding overall survival

Variables	Univariate			Multivariate		
	HR	95%CI	P-value	HR	95%CI	P-value
Age (years)	2.177	1.359-3.489	0.001	1.791	1.095-2.929	0.020
(≥65 vs. <65)						
sex	1.234	0.809-1.884	0.329			
(male vs. female)						
Tumor size (≥3 vs. <3)	1.328	0.581-3.036	0.501			
Tumor location						
Frontal lobe	0.654	0.431-0.993	0.046	0.770	0.476-1.245	0.285
(yes vs. no)						
Temporal lobe	0.993	0.665-1.485	0.974			
(yes vs. no)						
Midline structure	1.651	1.062-2.568	0.026	1.410	0.852-2.332	0.181
(yes vs. no)						
Mutilobar	1.226	0.807-1.862	0.339			
(yes vs. no)						
Other locations	1.538	1.029-2.299	0.036	1.251	0.821-1.906	0.297
(yes vs. no)						
Extent of resection	0.242	0.157-0.375	<0.001	0.264	0.168-0.416	<0.001
(Gross total vs. Subtotal)						
Adjuvant radio/chemotherapy	0.344	0.227-0.521	<0.001	0.414	0.268-0.640	<0.001
(yes vs. no)						
Triglycerides	0.858	0.577-1.267	0.451			
(≥1.24 vs. <1.24)						
Total cholesterol	0.508	0.302-0.855	0.011	0.677	0.305-1.501	0.337
(≥3.91 vs. <3.91)						
HDL cholesterol	0.662	0.445-0.986	0.043	0.686	0.451-1.045	0.079
(≥1.32 vs. <1.32)						
LDL cholesterol	0.376	0.198-0.715	0.003	0.412	0.211-0.801	0.009
(≥1.84 vs. <1.84)						

HDL, high-density lipoprotein; LDL; low-density lipoprotein.

### Relationship between pre-surgical serum LDL cholesterol level and other clinical characteristics

The LDL cholesterol level was associated significantly with total cholesterol levels (P<0.001). None of the other clinical parameters was associated with LDL cholesterol <1.84 mmol/L, including age at surgery, sex, tumor location, tumor size, triglycerides level, and HDL cholesterol level (Table 3).

**Table 3:** Relationship between pre-surgical serum LDL cholesterol level and other clinical characteristics

Variables	LDL cholesterol ≥ 1.84 mmol/L	LDL cholesterol < 1.84 mmol/L	P
Age (years)			0.056^b^
≥65	19	5	
<65	95	6	
sex			1.000^b^
Male	74	7	
Female	40	4	
Tumor size (cm)			
≥3	106	10	0.576^c^
<3	8	1	
Tumor location			
Frontal lobe	46	3	0.599^b^
Temporal lobe	43	7	0.176^b^
Midline structure	32	1	0.315^b^
Mutilobar	37	1	0.206^b^
Other locations	43	4	1.000^b^
Triglycerides			0.330^a^
≥1.24 mmol/L	59	4	
<1.24 mmol/L	55	7	
HDL cholesterol			0.675^b^
≥1.32 mmol/L	65	5	
<1.32 mmol/L	49	6	
Total cholesterol			<0.001^b^
≥3.91 mmol/L	103	3	
<3.91 mmol/L	11	8	

HDL, high-density lipoprotein; LDL, low-density lipoprotein; a: Pearson Chi-Square Test; b: Continuity Correction; c: Fisher's Exact Test.

## DISCUSSION

Glioblastoma remains a highly lethal malignant tumor with an unfavorable prognosis. Although patients with glioblastoma receive combined radiotherapy and chemotherapy following surgical resection, the final clinical outcomes are diverse [10], suggesting that other variables may influence survival and disease prognosis. Therefore, identifying the potential prognostic factors might guide individual therapy.

Cholesterol, as a main component of the cell membrane, plays an essential role in tumor cell growth and proliferation. It is derived either from receptor-mediated uptake of LDL from circulating blood, or by *de novo* synthesis [6]. The LDL receptor has been shown to be highly expressed in glioblastoma and other types of cancers [11–13]. Therefore, the proliferation and growth of glioblastoma cells might lead to uptake of cholesterol from the blood and result in a decrease in serum cholesterol level. In our study, low serum LDL cholesterol level remained an independent prognostic factor for poor OS in patients with glioblastoma. Thus, rapidly growing glioblastoma cells might take up circulating LDL cholesterol from the blood at a high rate, resulting in lower serum LDL cholesterol level. Based on previous studies, glioblastoma was classified as a small (size<3 cm) or large (size≥3 cm) tumor in the present study [14, 15]. Rapidly growing glioblastomas may be larger in size, but we did not find any association between low serum LDL cholesterol levels and a large tumor at the time of surgery. This could be attributed to several aspects. First, Stensjøen et al. found that large glioblastomas had significantly lower growth rates compared with smaller ones, supporting the assumption that large tumors reach a plateau phase with slower growth rates and thus follow the Gompertzian growth model [16]. Besides, glioblastoma is a heterogeneous cancer, both in terms of patient prognosis and molecular profile [17]. Therefore, these may explain why serum LDL cholesterol levels were not associated with tumor size in our study.

Patients with potential comorbidity affecting lipid profiles were excluded from our study, including diabetes mellitus, chronic lymphocytic leukemia, and cerebral infarction. Stamouli et al. have reported that 70.0% of patients with diabetic mellitus present with at least one lipid abnormality; and increased total cholesterol, increased LDL cholesterol, increased triglycerides, and reduced HDL cholesterol levels were noted in 36.37%, 28.37%, 39.01%, and 30.12% of the patients, respectively [18]. Yavasoglu et al. observed low LDL, HDL, and total cholesterol levels in patients newly diagnosed with chronic lymphocytic leukemia [19]. In the present study, two patients with a history of cerebral infarction and on aspirin and statin medications were excluded. Yang et al. have suggested that aspirin could inhibit abnormal lipid metabolism in hepatocellular carcinoma cells [20]. Kotani et al. have observed that in older people, serum total cholesterol metabolism alterations could be related to aspirin metabolism [21]. Moreover, Statins (inhibitors of the 3-hydroxy-3methylglutaryl coenzyme A) are very potent lipid-lowering drugs and therefore are widely used in western countries [22].

In literature, lower serum cholesterol level is reported to be associated with suppressed immune function. A study by Muldoon et al. [23] found that individuals with low cholesterol level have significantly fewer CD8+ cells, total T cells, and circulating lymphocytes, than those with high serum cholesterol level. Low serum cholesterol level may be related to a low serum antioxidant reserve, probably increasing susceptibility to oxidative stress [24]. C-creative protein (CRP) and interleukin-6 (IL-6) are well-known markers of systemic inflammation. It is recognized that the inflammatory response is closely associated with disease progression in patients with cancer [25]. An increased serum interleukin-6 level has been linked to a lower serum total cholesterol level in patients with prostate cancer [26]. In addition, a study found that the serum CRP level changes were significantly inversely correlated with the serum total cholesterol level in patients with clear cell renal cell carcinoma [5]. Nevertheless, the detailed mechanisms underlying the association between low serum cholesterol level and systemic inflammatory responses in patients with glioblastoma require further investigation.

Several oncogenic signaling pathways, such as receptor tyrosine kinase (RTK)/Ras, phosphatidyl-3-kinase/protein kinase B (PI3K/AKT) and tumor protein 53 (TP53) pathways have been found to regulate cholesterol synthesis in cancer cells [27]. In the recent past, statins has been shown to inhibit the growth of glioblastoma cells [28, 29]. However, the results of clinical studies are controversial. A recent study did not reveal a link between preoperative statin use and favorable survival prognosis in patients with glioblastoma [30]. In contrast, a different study revealed that pre-diagnostic long-term statin use correlated with favorable prognosis following glioblastoma diagnosis [31]. This inconsistency could be explained by several aspects: first, the different duration, dose, and type of statins used; second, the different clinical conditions of patients at the time of surgery, and finally, the different percentage of patients in the statin group received adjuvant treatment (radiotherapy and chemotherapy). Owing to the observational nature of these studies, further larger prospective randomized clinical trials are needed to resolve the issue and validate these findings.

To the best of our knowledge, this is the first study to investigate the prognostic value of pre-surgical serum lipid levels in patients with glioblastoma. The results of this study suggest that LDL cholesterol level is an independent prognostic factor for OS in patients with glioblastoma. However, the present study has some limitations. Firstly, the above analyses are based on data obtained from a single tertiary hospital in West China. Secondly, selection bias was unavoidable owing to the retrospective nature of the study. Thirdly, the lipid levels after the tumor resection were not measured in these patients. Furthermore, information on body mass index (BMI), which correlates with lipid profiles, was not routinely recorded in our medical records. Therefore, the patent's pre-surgical BMI was not taken into consideration in this study. Despite these limitations, this study clearly indicates that pre-surgical serum LDL cholesterol level might provide additional significantly useful information for clinical management of patients with glioblastoma. Our results could therefore serve as a useful benchmark for future research. Further studies with larger sample size are required to confirm our findings.

## MATERIALS AND METHODS

### Clinical data collection

A total of 153 patients with histopathologically confirmed glioblastoma (as per the 2007 World Health Organization brain tumor classification) who underwent surgical resection between January 2015 and May 2016 at the West China Hospital were enrolled in the study. Fasting serum lipid levels (total cholesterol, HDL cholesterol, LDL cholesterol, and triglycerides) were measured in patients as part of routine pre-surgical assessments at the Department of Clinical Laboratory. Patient age and sex; tumor size (≥3 cm and <3 cm); tumor location; extent of tumor resection (subtotal resection or gross total resection); adjuvant treatment (radiotherapy and chemotherapy); and pre-surgical serum lipid levels were determined by reviewing medical records. Pre-surgical magnetic resonance imaging scans and operation notes were examined to determine the location and size of the tumor. Infiltration of the frontal or temporal lobe, midline structure or other sites, was assigned. For statistical considerations, tumors extending over multiple lobes were assigned multiple counts. Seven patients with a prior history of intracranial glioma surgery, three with diabetes mellitus, two with a history of cerebral infarction, and receiving aspirin and statin treatment, one with a history of radiotherapy prior to surgery, and one patient with chronic lymphocytic leukemia were excluded from the study. Four patients who died shortly after the operation, owing to serious post-surgical complications were also excluded from the study. Serum lipid levels were not determined in one patient, and nine patients were lost to follow-up. Finally, 125 patients with glioblastoma were included in this study. OS was calculated as the time between surgical resection and the date of death, or the date of last follow-up. The last follow-up was performed in May 2017.

### Statistical analysis

The X-tile software (Version 3.6.1, Yale University) was used to determine the optimal cut-off values for serum lipid levels [32], as applied previously [33–35]. Statistical analysis was performed using SPSS software (version 19.0). A P-value of less than 0.05 was considered statistically significant. The chi-square test was used to examine the relationship between lipid levels and clinical parameters. Survival curves were acquired using the Kaplan-Meier method. Univariate and multivariate statistical analysis were performed using the Cox proportional hazards regression model. In univariate analysis: age; tumors infiltrated the frontal lobe, midline structure, other locations; extent of resection; adjuvant radio/chemotherapy; total cholesterol, HDL cholesterol, and LDL cholesterol levels were all found to be significant prognostic factors. These nine significant variables were then entered into a multivariate model. At the last follow-up, 27 patients were still alive and 98 were dead. According to the principles of multivariate regression analysis, the number of clinical events (e.g. deaths) should be at least 10 times the number of candidate variables for prognosis that are included in the multivariate model [36, 37]. Therefore, the sample size in this study meets the minimal requirements for statistical analysis.

## Abbreviations

OS: Overall survival
HDL: high-density lipoprotein
LDL: low-density lipoprotein
IGFBP-2: Plasma insulin like growth factor binding protein 2
CRP: C-creative protein
IL-6: Interleukin-6
RTK: Receptor tyrosine kinase
PI3K/AKT: Phosphatidyl-3-kinase/protein kinase B
BMI: body mass index
